# Football Match Dynamics Explored by Recurrence Analysis

**DOI:** 10.3389/fpsyg.2021.747058

**Published:** 2021-09-24

**Authors:** Martin Lames, Sebastian Hermann, René Prüßner, Hendrik Meth

**Affiliations:** ^1^Faculty of Sports and Health Sciences, Technical University of Munich, Munich, Germany; ^2^Department Big Data and Data Science, Hochschule der Medien, Stuttgart, Germany; ^3^Kinexon GmbH, Munich, Germany

**Keywords:** recurrence analysis, football (soccer), complex systems, performance indicators (PIs), recurrence parameters

## Abstract

A widely accepted notion of football matches in performance analysis (PA) is to consider them as dynamic interaction processes with emerging behaviors. The description and analysis of these processes requires specific methods. Recurrence analysis is a technique for analyzing complex systems in many domains like astrophysics, earth sciences, engineering, biology, cardiology, and neuroscience. Its general concept is to examine the recurrence behavior of a system, as in when, how often and how close its trajectory in a phase space returns to a previous state. The aim of the study is to apply recurrence analysis to football matches. Positional data from 21 football matches of a German Bundesliga team were examined. The phase space was made up of the field players' x,y-positions at each second of the match. For each pair of seconds, the average distance of all the players between their positions at these two time points was calculated. Recurrence plots (RPs) were obtained by color-coding these distances. With a recurrence threshold of rt = 9 m and a minimum line length of l_min_ = 3 s, general recurrence parameters were calculated to characterize the individual recurrence behaviors of each match. Three football-specific recurrence parameters were defined to represent recurrence properties of open play. RPs showed commonalities (typical features indicating set plays and continuous gameplay) as well as unique structures during each match (number, distribution, and sequence of typical features). The recurrence parameters showed several significant correlations with traditional performance indicators like number of goals and passes completed, e.g., the correlation between number of goals and recurrence rate is *r* = −0.622 (*p* = 0.003). By extending the sample and design of recurrence studies, there is great potential for recurrence analysis to improve both the practical and theoretical potential of performance analysis.

## Problem

A widely accepted approach to the performance analysis (PA) of football matches is to consider them as dynamic interaction processes with emerging behaviors (Gréhaigne et al., 1997; McGarry et al., 2014; see also Parlebas, 2020). The reason for this is that, on the one hand, there are tactical plans in place that drive collective behavior to successfully achieve a team's goals. On the other hand, this behavior can also be seen in the other team, so both teams are trying to prevent each other from reaching their goals. This leads to players frequently moving back and forth across the pitch, caused by within-team and between-team interactions (McGarry et al., 2002).

These interactions are governed by many influencing factors, including the general action plans of a team and single players, the specific match plans developed in preparation for a match, and the situational variables, such as score-line-dependent tactics and momentary positional configurations (with their constraints and affordances). In addition, mere chance effects are also present, for example, when goals in football are scored as a result of uncontrollable variables like deflections (Lames, 2018; Wunderlich et al., 2021). From a dynamical systems perspective, actual behavior is considered the result of the self-organization between all of these factors, which then leads to the final result—the perceivable behavior.

The description and analysis of these dynamic interaction processes, along with their emerging patterns requires specific methods. Traditional performance analysis mostly examines these aspects with respect to playing style (Fernández-Navarro et al., 2018; Gómez et al., 2018; Lago-Peñas et al., 2018; Castellano and Pic, 2019). Common performance indicators, e.g., such as ball possession or duels won (Sampaio and Leite, 2013), fail to meet these requirements because they provide only single figures that summarize a single aspect of the match (Lames and McGarry, 2015). Recent approaches in PA that reconstruct certain game aspects either by using machine learning techniques, such as the tactical line-up (Bialkowski et al., 2014), or by directly modeling certain constructs, such as the dominated region (Gonçalves et al., 2017), passing availability (Goes et al., 2021), or dangerousity (Link et al., 2016), are not designed for this purpose either.

Kuznetsov et al. (2014) introduced non-linear time series methods with the potential to analyse complex systems in sport. Besides fractal measures like spectral analysis, these methods propose entropy measures like Approximate Entropy (Pincus, 1991) and Sample Entropy (Richman and Moorman, 2000), which have already found applications in the dynamic analysis of order and predictability in football matches (Fonseca et al., 2012; Sampaio and Maçãs, 2012; Duarte et al., 2013; Barnabe et al., 2016). While these entropy measures describe the organizational state of a system with a single figure changing over time to express the dynamics of the system, Kuznetsov et al. (2014) suggest that recurrence quantification analysis (RQA; Eckmann et al., 1987) could also serve as an appropriate tool for non-linear time series analysis.

The general concept of RQA is to identify repeating patterns represented by the phase space trajectory, for example, when the trajectory returns to the present state at a later point in time. A phase space is typically a high-dimensional space that contains all possible states of a complex system. The central tool for examining the recurrence behavior of a time series is the recurrence plot (RP). An RP is a nxn-pixel matrix with n representing the length of the time series being investigated. Each pixel has a color value that corresponds to the distance between the positions in phase space for each pair of time points t_x_ and t_y_. A dichotomous RP introduces a black pixel at point (t_x_,t_y_) whenever the distance between the positions in phase space is smaller than a certain recurrence threshold. This procedure creates a pixel pattern that is distinctive of the examined time series. Thus, in RQA, the dynamics of the underlying complex system is expressed in a two-dimensional RP with typical patterns formed by these “recurrence points” characterizing the complex dynamics of the original system. This gives RQA a conceptual advantage compared to other non-linear time series methods like Approximate Entropy or Sample Entropy that only express the current state in a single figure.

A method already known for analyzing recurrent structures in sports science is the T-patterns approach. This approach analyzes time series data by searching for recurring episodes (Magnusson, 2000, 2020). The T-patterns approach has been applied to detect and describe recurring sequences of behavioral events in team sports such as soccer (Borrie et al., 2002; Jonsson et al., 2010; Camerino et al., 2012). Interesting findings from the T-patterns approach are that the number of significant patterns has a high correlation with experts' assessments of team quality (Jonsson et al., 2010) as well as winner-loser differences in boxing (Pic and Jonsson, 2021). In contrast to recurrence analysis, where any phase space may be investigated, the T-patterns approach is based on sequences of categorical event data, thus it focuses primarily on the identification of typical plays (Gudmundsson and Wolle, 2014). The aim of recurrence analysis is to provide a comprehensive overview of recurrent (and non-recurrent) structures in a match.

In addition to the qualitative patterns in an RP, RQA also allows for the measurement of objective features of the RP by so-called recurrence parameters. These parameters are calculated from the pixel matrix and represent certain aspects of it. For example, the general degree of recurrence is assessed by the parameter “recurrence rate” and is defined as the number of recurrence points expressed as a percentage of the total number of points in the RP. Using recurrence rate as an example of a recurrence parameter, it is possible to observe a high correspondence between this parameter and the practical notions of football matches. When the recurrence rate is high, we expect a very structured game with many similar ball possession periods with respect to the positional configurations of offensive and defensive plays. On the other hand, when the recurrence rate is relatively low, we expect a very chaotic game with many interruptions and many short ball possessions with frequent turnovers, where it is unfeasible for stable, repeating positional patterns to evolve.

For several decades, performance analysts dispose of positional data of football players who were tracked for the duration of a match. Thus, a convenient and appropriate phase space for football is the x,y-coordinates of all the players over time, containing a complete spatio-temporal representation of the match. When doing so, it is very likely that tactical patterns such as position attack or fast attack are repeated in a similar manner over the duration of a match, leading to corresponding patterns in the RP. Moreover, set plays like corners and free kicks, which typically show a unique and similar positioning of the two teams, are expected to appear in RPs as well. Recurrence parameters from the RP, such as the aforementioned recurrence rate, could then be validated and used as performance indicators in football.

RQA, although acknowledged as a tool for non-linear time series analysis (Kuznetsov et al., 2014), has only found very few applications in PA so far. Cotuk and Yavuz (2007) investigated the time series made up by the number of contacts in ball possessions in football with an RP. Carvalho et al. (2013) examined the recurrence rate and maxline (longest parallel trajectory of consecutive recurrent points) in tennis, where they found significant differences in both the recurrence parameters before and after a break shot (i.e., a perturbation shot in tennis rallies).

So far, Stöckl et al. (2017) have provided the most comprehensive introduction to RQA in sports. They describe, in detail, the methodological steps to arrive at a solution. Two applications of RQA are presented. First, sequences of shots by 74 golfers were analyzed over four rounds in a golf tournament. For each shot, its quality was assessed by the Shots Saved statistic (Stöckl et al., 2012). It could be demonstrated with RQA that even top-class golfers showed unpredictable or chaotic behavior regarding the quality in a series of shots.

The second application of RQA, and the reference to the present paper, is a study on football based on Lames and McGarry (2015). Twelve matches from the 2009 to 2010 Bundesliga season were analyzed with RPs and RQA. A qualitative identification of match events was done, and these events were correlated with the number of recurrence points at the time of the event; this is known as the pointwise recurrence rate. Results show that the highest number of recurrence points occurs during open play, whereas special and rare situations like corners and free kicks, as well as shots on goal, show a lower number of recurrence points.

This observation is ascribed to the fact that, on the one hand, in open play situations, typical defensive, and offensive configurations are found and occur repeatedly throughout a match, with the exception of shots on goal, which are only delivered on rare, singular occasions. On the other hand, set plays are rare events, which explains the lower recurrence rates for set plays compared to open play. Within set plays one may find a high recurrence, because the positions of players before a set play, i.e., waiting for a corner, are quite static, and as such, are locally recurrent. Moreover, the configurations between two similar set plays, e.g., corner from the left side for team A, should also be quite similar leading to high recurrence rates between similar set plays.

Stöckl et al. (2017) also investigated correlations between recurrence parameters and typical football performance indicators and revealed several significant relationships. In addition, they presented interesting interpretations of qualitative features of the football RPs with the identification of “blocks” in the RP, i.e., rectangular areas with a higher density of recurrence points and clearly defined boundaries marked by broader white bands.

These football-specific recurrence structures may be associated with open play phases without long interruptions, meaning they consist of alternating sub-phases with highly repetitive structures and medium to low repetitive structures. This pattern corresponds to the phenomenon of “metastability” (Kelso, 2012) that has been previously discussed in dynamical systems theory. Metastability expresses the idea that the system is not characterized by a few stable states with very energy-demanding transitions between them, but by many stable states with easier and more frequent transitions. Originally, metastability was introduced as an extension of bi- or multi-stable systems for example in coordination dynamics (Kelso, 1995). Later, it was discovered that metastability is the rule rather than the exception in complex systems because, in biological systems like the brain, it provides evolutionary advantages (Kelso, 2012). Recently, the concept of metastability was also successfully applied to describe self-organization processes leading to collective behavior (Tognoli et al., 2020).

In football, metastability is an interesting concept for describing the distinctive back-and-forth movement of football-specific collective behavior. The “blocks” identified by Stöckl et al. (2017) would then represent longer phases of these back-and-forth movements or the “to-and-fro behavior” as described by McGarry et al. (2002). The internal structure of these blocks, an alternating pattern of highly recurrent periods and short periods of lower recurrence between them, may be connected to the collective behavior occurring during these phases. Sub-phases of open play consist of controlled ball possessions with a higher degree of order and recurrence. These sub-phases typically only last a relatively short amount of time (the average duration of a ball in play was found to be 32 s by Siegle and Lames, 2012), and a perturbation is likely to happen. After a perturbation, there is a less-controlled and less-structured sub-phase with lower recurrence values, but after some time, when one of the teams establishes stable ball possession, there will be higher recurrence again. Thus, metastability in football may be represented in an RP of a football match.

These considerations lead to the aims of the study. The first aim is to conduct an RQA on football matches, substantially extending upon the rather small sample investigated so far. Secondly, suggestions will be made to calculate new recurrence parameters specific to football by investigating the properties of the “blocks” in the RPs, as explained above. Thirdly, for validation purposes, correlations between the recurrence parameters and traditional performance indicators will be calculated to demonstrate the capability of RQA, not only to confirm existing knowledge, but also to add new insights. The general aim of the present study is thus to explore the potential contributions of recurrence analysis to football theory and to suggest further studies in performance analysis using this new tool.

## Methods

### Sample

The sample consists of 21 matches of a single club in the 2019–2020 season of the German Bundesliga. Position data was recorded at a sampling rate of 25 Hz for all players from both teams on the pitch during that specific match, each one identified by a unique player ID. The data was collected by TRACAB, the official provider of position data for the German Bundesliga 2019–2020 season. The system used was the TRACAB Gen4, a stereo camera system consisting of two multi-camera units in two locations on either side of the halfway line, each consisting of three HD-SDI cameras with a resolution of 1,920 × 1,080 pixels.

In a recent validation study (Linke et al., 2020) using the VICON system as the gold standard the accuracies of the position, velocity, and acceleration data were tested performing a small-sided game on a 30 m × 30 m pitch and a football-specific course. The pooled RMSEs were 0.09 m for position, 0.09 m/sec for velocity and 0.26 m/sec^2^ for acceleration, thus showing very acceptable accuracy in absolute standards, as well as in relation to other position detection systems (Linke et al., 2018). Especially for the purpose of this study, the accuracy of the positional data with an average error of 9 cm is satisfactory.

### Recurrence Analysis

Recurrence analysis is a technique for analyzing complex systems in many domains like astrophysics, earth sciences, engineering, biology, cardiology and neuroscience (Marwan et al., 2007). The general concept has been explained in the problem section of this paper. Here, the methodological considerations before conducting a recurrence analysis in football are given in detail.

The phase space representing a football match is comprised by the x,y-positions of the players for each second of the match. As goalkeepers usually play on a limited area on the pitch and tend to stay in the same place (in front of the goal) for the majority of the match, they were eliminated from the phase space to avoid recurrence inflating effects. Thus, the football matches are represented by a trajectory in a 40-dimensional phase space (2 teams, 10 players per team, 2 spatial coordinates).

The length of the matches was fixed to 2*45 min = 90 min exactly, because otherwise, recurrence parameters could differ due to different match lengths. Data was down-sampled from the raw data collected at 25–1 Hz, which—besides computer running time problems, see below—is sufficient for capturing the essential movements of the players on the pitch. At each second, the Euclidean distance between the positions of the 20 players and their positions at any other second was calculated. Thus, our RPs contain (90*60)^2^ = 5,400^2^ = 29,160,000 points. Further data processing steps are described below.

Usually, the first step in recurrence analysis is to embed the time series under scrutiny into a higher dimensional phase space by selecting an appropriate embedding dimension and delay vector (Marwan et al., 2007). While there is a discussion in dynamical systems theory about whether embedding is necessary for each time series in the analysis (Iwanski and Bradley, 1998), the situation in our study is different from typical recurrence studies. The reason for using embedding techniques is typically because the complete phase space of a complex dynamic system is not known. Instead, one only has a one-dimensional time series indicating an outcome of the complex system, e.g., the temperature curve over some period indicating the outcome of the complex dynamical system weather. In football, we are quite confident that the spatio-temporal behavior of the players is contained in the 40-dimensional phase space specified above. This leads us to the decision to omit embedding in our recurrence analysis of football matches, i.e., the applied embedding dimension is 1 and the delay vector has a length 1.

### Data Processing

The collected raw data needed extensive pre-processing, including data reduction, data transformation and the generation of new features, before RPs could be drawn and recurrence parameters could be calculated.

First, the data was reduced to only the features relevant for the RP calculation. The remaining feature set consisted of identifier fields for the game, the player and the team as well as a timestamp (in ms) and the position data (x in m and y in m). In the second step, goalkeepers were detected based on their specific movement patterns and deleted from the data set (as explained above). Furthermore, the data was down-sampled from 25 to 1 Hz to enable visualization in an appropriate format (5,400 × 5,400 pixels). Finally, the time period per game was normalized to exactly 5,400 s (2,700 s for each half), resulting in a standardized format for each game and allows for comparisons. Overtime periods were therefore removed from the data set. A transformation was required to “mirror” the position data from the second half to correct for the teams switching sides.

Due to player substitutions, it was also necessary to generate “player streams,” which represent mappings of position data from a player entering the field to a player leaving the field. This ensures we have comparable data for each of the 10 field players per team for the entire game. This step could not be fully automated, as in the case of double substitutions, as it cannot be unambiguously observed from the data which player is replaced by whom. Finally, dismissals following a red card were detected in the data to provide the number of players per team for each second of the game for the subsequent RP calculations.

The data was processed in multiple steps using a deep learning server equipped with 128 GB main memory, 4 NVIDIA RTX2080 GPUs and an i7 CPU (10 core). All calculations and diagrams were generated using Python and corresponding frameworks. Processing time was 11 min per match for calculating the RP, i.e., around 4 h for the 21 matches.

### Recurrence Thresholds

A critical decision in recurrence analysis is to define the recurrence threshold (rt), i.e., the threshold below which the distance between two points in the phase space must fall in order to identify a recurrence point. A non-ambiguous definition for recurrence points is critical for calculating recurrence parameters. As we have the positional data for all of the players, it is necessary to specify the threshold as a certain extent of mean player distance. There are several methods for the calculation of rt. In this study, the recommendation by Marwan et al. (2007) was applied, and a recurrence threshold maximizing the mean of diagonal lines (recurrent paths) in relation to the entropy of line lengths was chosen. The rt was set to a mean difference of 9 m for all 20 players. This threshold was applied and has been discussed already in Stöckl et al. (2017). While 9 m per player may seem quite high in relation to the pitch dimensions (usually 68 m wide and 105 long), the players' configurations tend to remain quite stable (i.e., strong in-phase coupling; Siegle and Lames, 2013), and since players tend to stay in the central area of the pitch, a distance of 9 m can be considered tactically equivalent in most cases.

A convention on the shortest diagonal line length (l_min_) is required to calculate recurrence parameters. Here, a compromise was sought for balancing short minimum lengths resulting in more recurrence points, while disguising recurrence structures, and long minimum lengths with only a few recurrence points. Again, we adopt the suggestion by Stöckl et al. (2017) specifying l_min_= 3. This means we only refer to a recurring episode when players stay below the recurrence threshold for at least 3 s.

### Recurrence Plots

The calculated 5,400 × 5,400 data points were visualized in a heat map, color-coded with the corresponding distances (see **Figure 2**). The maximum distances per player were relatively consistent across all games with values in the range of [80;90] meters. The selected color map displays small distances in shades of red (dark red to orange), medium distances in shades of green (lime green to turquoise) and large distances in shades of blue (light blue to violet). To achieve a standardized, comparable color spectrum, distances for coloring were truncated to a maximum of 70 m, resulting in a color spectrum evenly distributed at distances between 0 and 70 m.

### Recurrence Parameters

As already mentioned, recurrence analysis does not only provide RPs as a qualitative representation of recurrence patterns, but also allows for the calculation of properties of the RP, and thus, the execution of recurrence quantification analysis (RQA). There are suggestions in the literature for some general recurrence parameters, seven of which will be used and are explained below. Moreover, for each type of process under consideration, specific parameters were defined to quantify the meaningful patterns observed in the RP. Thus, in addition to the seven general recurrence parameters, we also suggest three extra football-specific recurrence parameters that aim to characterize properties of the “blocks” in the football RPs that are associated with open play, as explained in the introduction.

#### General Recurrence Parameters

**Figure 1:**
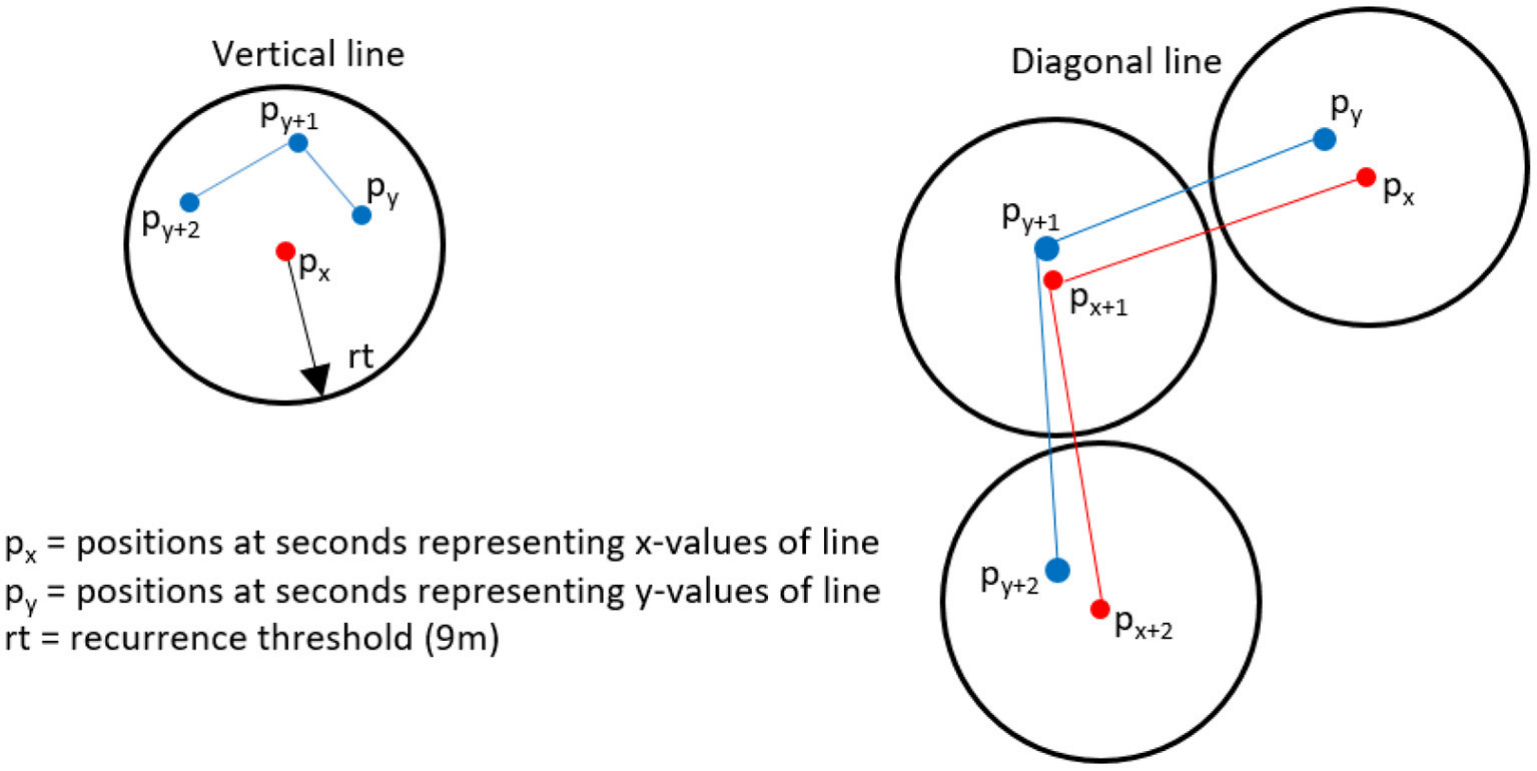
Illustration of trajectories resulting in vertical and diagonal lines; a vertical line is created when the process after time t = y stays in the rt-vicinity of a point p_x_, i.e., for t = y+1, y+2,…; a diagonal line is created when the process unfolds within the rt-vicinity after time t = x and t = y, i.e., stays below rt at t = x+1, t = y+1; t = x+2, t = y+2;.

Marwan et al. (2007) suggested the use of general recurrence parameters to quantify certain aspects of recurrence. Most of them are based on the analysis of diagonal and vertical “lines” in the RP, i.e., adjacent recurrence points that form either diagonal or vertical/horizontal (equivalent because of symmetry) lines with a length of at least l_min_, a parameter fixed by convention. A diagonal line means that the process unfolds for a certain period of time (the length of the diagonal line) in a similar, recurrent manner, whereas a vertical line indicates that the process stays “trapped” for a certain period of time (the length of the vertical line) in the vicinity of an initial point given by the recurrence threshold (see Figure 1).

These notions can easily be transferred to football, as diagonal lines here indicate that the game unfolds in a recurrent manner over a certain time span, i.e., with player configurations that differ less than an average of 9 m (the recurrence threshold). The diagonal line length corresponds to the time at which the game unfolds in this recurrent manner. Analogously, vertical lines mean that the configuration on the pitch differs <9 m per player on average from the initial configuration. The diagonal lines represent dynamically recurrent sequences, for example, when both teams move across the pitch in a repeating sequence, whereas vertical lines could mark static events where a configuration is maintained for a longer period of time, for example, when teams are waiting for a corner. In short, vertical lines represent static recurrence and horizontal ones dynamic recurrence.

We adopted seven recurrence parameters from Marwan et al. (2007):

1. Recurrence Rate (RR): This is the most well-known recurrence parameter and the only one that is not associated with lines in the RP. It simply counts the recurrence points in the whole RP and provides the rate of these points compared to all points in the RP. Essentially, RR quantifies the general degree of recurrence in the examined process. In football, we might expect to see a range of RR values that are typical for the sport with some variance due to very chaotic, unstructured matches (low RR) and very structured repetitive matches (high RR), for example, when a team exhibits a possession-oriented playing style.
2. Determinism (DET): DET is the ratio between the recurrence points that lie on a diagonal line of length l with l ≥ l_min_ (l_min_ = 3 in our case) and the total number of recurrence points. DET signifies the fraction of longer instances of recurrence compared to all recurring instances. If DET is comparably high, we predominantly have structured recurrent sub-processes. If not, then there is more random recurrence. In football, we might expect higher values of DET because football tactics are known to have certain preferred spatial configurations and pre-planned processes in both defense and offense.
3. Laminarity (LAM): LAM means very much the same as DET, but for vertical lines instead of diagonal ones. It is the fraction of all the recurrence points contained in vertical lines with length l ≥ l_min_ (l_min_ = 3 in our case). The semantic difference in football is that diagonal lines represent periods where the game unfolds in a similar manner, whereas vertical lines represent periods where the initial configuration stays similar for a period of time. In football, DET could be more characteristic of open play, whereas LAM is high when there are many set plays.
4. Average diagonal line length (LL): LL is defined as the average of all diagonal lines with l ≥ l_min_. LL is also called the “mean prediction time,” indicating that the process, on average, will unfold for this duration of time once it is in a recurrent state.
5. Trapping Time (TT): TT is analogous to LL but is the average vertical line length of all lines with l ≥ l_min_. TT is the average time a match configuration remains close to an initial configuration.
6. Entropy (ENTR): ENTR is the Shannon entropy of the different diagonal line lengths ≥ l_min_. Frequently-occurring line lengths contribute largely to ENTR, while rarer line lengths contribute less to ENTR. This results in low ENTR values that represent chaotic systems with few perceivable and mostly short patterns, whereas high ENTR values are characteristic of predictable, highly structured behavior. This means that more organized play in football should lead to higher ENTR values and vice versa.
7. ENTR-V: ENTR-V is the entropy of the vertical lines. Although not explicitly suggested by Marwan et al. (2007), ENTR-V is an interesting parameter in addition to ENTR because diagonal and vertical lines each represent different aspects of a football match as mentioned above.

For all of the aforementioned parameters, higher values are characteristic of a more structured, ordered or predictable behavior, whereas lower values stand for the opposite, namely a chaotic, random or unpredictable process. Nevertheless, all seven parameters focus on different aspects. On the one hand, they deal with either diagonal or vertical lines (except RR), both of which have unique meanings in football. On the other hand, the mean line lengths (LL and TT), the proportions of lines with length ≥l_min_ (DET and LAM) and the entropy of line lengths represent different aspects of the recurrent patterns under scrutiny. Thus, the recurrence of football matches is explored using these seven general recurrence parameters, as proposed in the literature. In addition, we also suggest three football-specific recurrence parameters.

#### Football Recurrence Parameters

As mentioned previously, football is characterized by longer phases of open play with more organized phases and transitions between them. These phases could be characterized as phases of metastability (Kelso, 1995), where the stable pattern is the continuous alternation of more or less organized play. Stöckl et al. (2017) observed “blocks” in football RPs that represent these phases. In the following section, we suggest football-specific recurrence parameters based on these structures that represent what is called open play in football.

**Figure 2:**
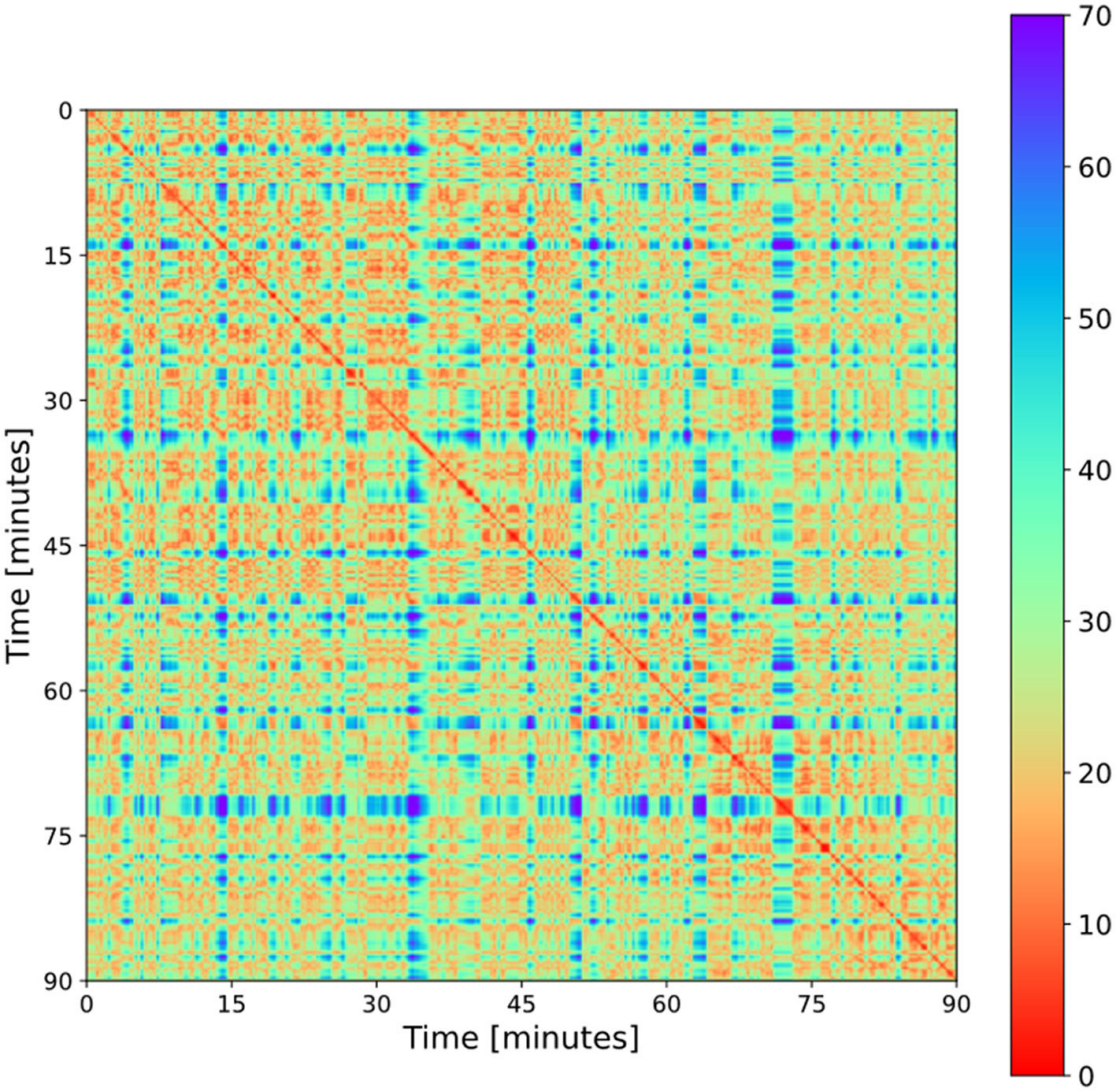
An RP of a football match (game 05). Typical features are the red diagonal stripe, blue/turquoise stripes or bands and rectangles or blocks containing red/orange spots embedded in green (explanations are given in the text).

The first task is to identify these “blocks.” The patterns perceived as blocks are areas with middle to high recurrence, separated by larger phases or events of low recurrence, i.e., interruptions or set plays (see Figure 2). As we expect to see high, but also middle recurrence in open play, we introduce a lower recurrence threshold of 18 m specifically for detecting these phases. Each point in time with a pointwise recurrence rate of RR ≥0.20 is used to represent open play.

In addition, the minimum duration of a phase of open play was set to 45 s. This assumption was informed by the average time interval between two game interruptions (around 32 s; Siegle and Lames, 2012). Finally, the gap between two blocks must be at least 45 s in order to detect distinct phases of open play. With this procedure, based upon some assumptions about the nature of the phenomenon under scrutiny, we are able to observe the block-like patterns in football RPs. In **Figure 4**, the applied pattern detection algorithm is shown.

As this pattern detection procedure represents the open play or metastable phases of the match, these areas of the RP may be used to define new football-specific recurrence parameters (FRPs):

1. Prevalence of open play (FRP-1): This is the overall area of the metastable regions in the RP, expressed as a percentage of the area of the whole RP. This parameter assesses the degree of open play in a match.
2. Recurrence rate of open play (FRP-2): This is the recurrence rate within the areas of open play. FRP-2 assesses whether open play is characterized more by stable periods of position attacks or more by “chaotic” transition phases between stable ball possessions.
3. Recurrence share of open play (FRP-3): This parameter is the fraction of recurrence points occurring in open play relative to all recurrence points. Aside from open play, recurrence points can also be found in recurrent set plays. If FRP-3 is relatively high, this indicates a more organized playing style with more structured attacks like FRP-1, but not compared to non-open play phases in the match but to other highly recurring points in the match.

### Traditional Performance Indicators

A number of traditional football performance indicators (PIs) were collected from the internet (www.kicker.de) for the 21 matches. As PIs are typically reported on a team basis, e.g., distance covered for team A and team B, these statistics had to be transformed because recurrence is reported on a match basis: either the sum or the mean was taken, e.g., the sum of corners of A and B or the mean distance covered by the two teams, the latter being identical to the mean distance covered by the 22 players on the pitch. The difference between the PIs of the two teams could also be taken to characterize a balanced match or a match dominated by one team, e.g., goal difference or difference in percent of duels won. A variable called “stoppages” was computed as the sum of goal, corner, hand, foul, and offside events.

As numerous PIs are routinely reported for each match in professional football, a sample of PIs was selected. They were chosen to represent the relationship between open and set plays (goals, shots on goal, pass statistics, corners, stoppages) and a team's degree of dominance (goal difference, distance covered difference, possession difference, duels won difference).

### Statistical Treatment

Recurrence parameters are given with the usual descriptive statistics, i.e., mean, standard deviation, maximum, minimum, and coefficient of variance (the percentage of the standard deviation relative to the mean).

Deviations from normal distribution were checked with the Kolmogorov-Smirnov-Test and no highly significant deviations were found, except for FRP-3, although the distribution of this variable is mono-modal and rather symmetric.

As a consequence, Pearson-Bravais correlations (*r*) were used to examine the relations between and within recurrence parameters and PIs. According to Cohen (1988), the effect size is small when *r* = 0.1, medium when *r* = 0.3 and strong when *r* > 0.5. In this study, it is also of interest whether we can reject the null hypothesis of *r* = 0. The thresholds for *n* = 21 matches are *r* = 0.433 for *p* < 0.05 and *r* = 0.549 for *p* < 0.01.

## Results

### Qualitative Features of RPs

Figure 2 depicts a color-coded RP for a selected match. There are some striking features that occur in each RP of a football match. First, there is a red diagonal stripe which is typical of RPs, as the average distance of the players at time t_x_ and t_y_ with x = y is zero, and is thus smaller than any recurrence threshold. It is interesting to see that the diagonal stripe is rather thin, suggesting that the player configuration will change imminently, i.e., the configuration on the pitch is highly dynamic. Nevertheless, some exceptions were found with some small squares appearing on the diagonal stripe. These squares characterize events where the players remain in nearly the same configuration for an extended period of time. For example, game stoppages where players are waiting for the execution of a penalty, free kick or corner result in these squares on the diagonal stripe.

A second striking feature is the blue/turquoise bands or stripes that are dispersed irregularly throughout the whole match. By definition, these are periods of low recurrence, i.e., rather unique events with a rare spatial configuration, such as set plays. Set plays often have a special lineup, for example, for corners or free kicks executed as crosses, we know that players with a strong header are positioned inside the penalty box. Typically, these are tall defensive players who are positioned in the first row, an area on the pitch where they almost never appear during open play. During these events, where a special lineup is used, these players are joined by other players, such as the one executing the corner or those safeguarding in the backfield, which is what produces these blue/turquoise bands or stripes. It was explained earlier that the red squares in the diagonal stripe frequently coincide with these bands. Moreover, it is interesting to see that we typically find blue crossings between two stripes, but occasionally red ones as well. This suggests that we typically have different set plays with different configurations (corner of team A and team B). However, for the same set play (corner of team A from the left side), a red crossing is very much to be expected when the same special lineup is used.

A third general feature of the football RPs are the “blocks” that have already been discussed in earlier sections of the paper. They represent phases of open play with embedded sub-phases of high recurrence (small red squares) interspersed with yellow or green areas marking the end of a highly structured position play, typically characterized by a perturbation with or without a change in ball possession.

**Figure 3:**
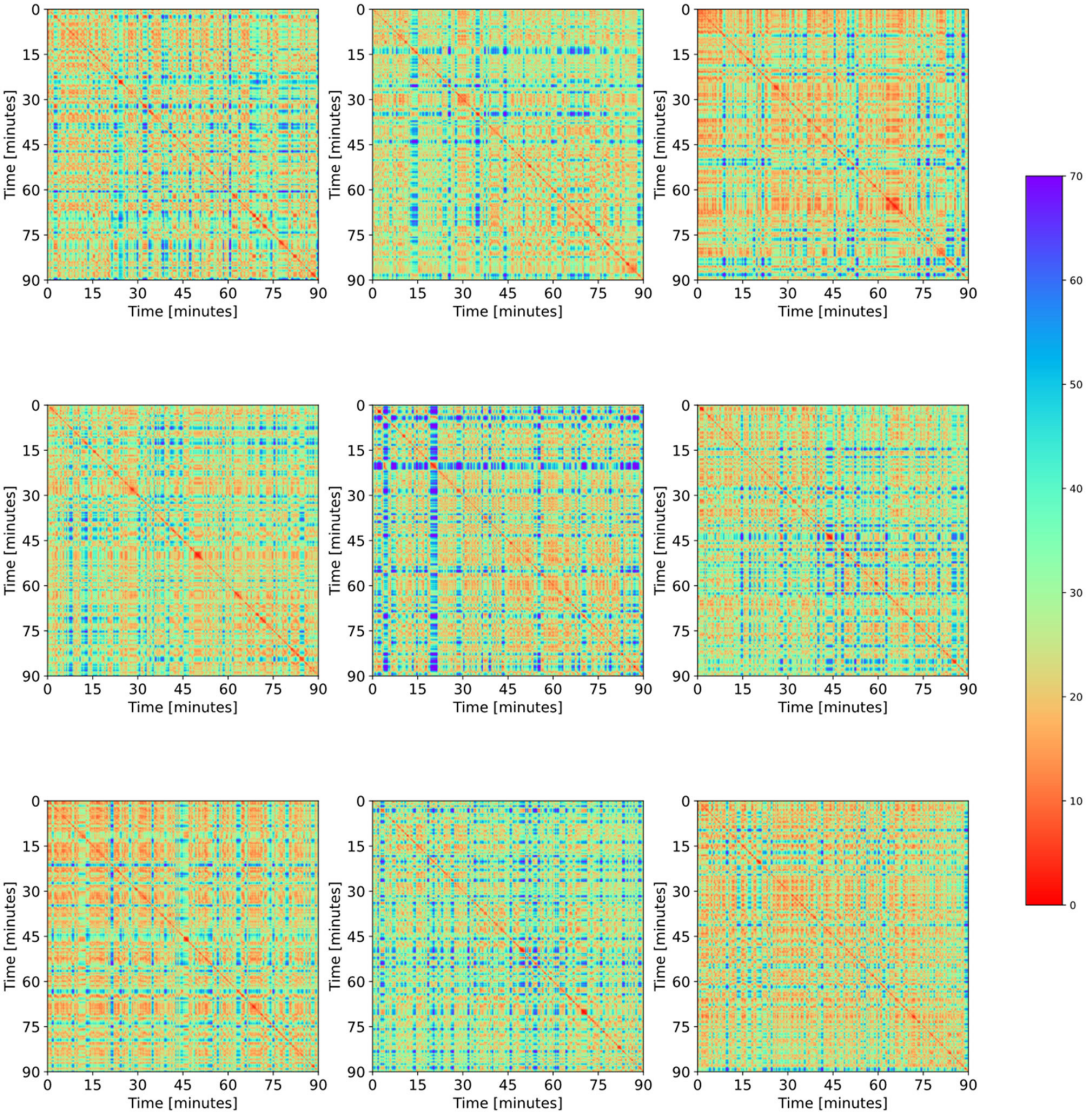
Thumbnails of 9 arbitrarily selected RPs. The three typical elements (red diagonal stripes, blue stripes, and red/yellow rectangles) can be found in each match, whereas their frequencies, sizes, and dispersions are highly match-specific.

Figure 3 shows RPs of 9 matches. This figure is provided to show that, on the one hand, all football RPs contain the three main features mentioned above. On the other hand, we observe that each RP can be seen as unique. The number of stripes or bands, their widths, their frequencies and their distributions over the whole match differ considerably from match to match. The same holds for the blocks of open play.

### Traditional RQA

Table 1 shows the descriptive statistics of the traditional recurrence parameters that were calculated according to Marwan et al. (2007). In general, football RPs show rather low recurrence rates when compared to other RPs, for example, RPs of periodical processes. The mean of RR is 0.015 with a span of 0.02. This means that, on average, 1.5% of the points in a football RP are recurrence points (range: 0.8–2.8%) with a relatively high coefficient of variation (34%), indicating that there are considerable differences between football matches with respect to recurrent events.

**Table 1:** Descriptive statistics of the 21 football matches, including the coefficient of variation (CoV) for traditional parameters of recurrence quantification analysis [RQA; Marwan et al., 2007].

**Parameter**	**Mean**	**STD**	**Min**	**Max**	**CoV**
RR	0.015	0.005	0.008	0.028	34.09
DET	0.867	0.017	0.837	0.904	2.01
LAM	0.933	0.009	0.917	0.951	0.96
LL	7.475	0.797	6.404	9.589	10.67
TT	8.468	0.693	7.605	10.360	8.18
ENTR	2.423	0.150	2.158	2.747	6.21
ENTR-V	3.171	0.141	2.924	3.504	4.43

Determinism and Laminarity show values around 90%, indicating that the length distribution of diagonal as well as vertical lines is shifted away from values below of l_min_ = 3. This suggests that there are longer recurrent periods caused by intentionally repeated behavior rather than chaotic or random recurrence. Also, relatively high values of determinism and laminarity at the same time produce a patch-like pattern made up of longer diagonal and vertical structures, as shown in Figures 2, 3. This pattern reflects the mechanical nature of movement on the pitch, including a certain latency imposed by the energy costs of changing positions very rapidly. It is interesting to note that for each football match, LAM was higher than DET, which suggests a higher prevalence of static phases compared to dynamic recurrent episodes.

The results for the average line length (LL) and trapping time (TT) show the same relationship as DET and LAM, i.e., the persistance of an initial state lasts longer than two episodes following the same track. Moreover, the average duration of episodes that unfold in a recurrent manner during a football match is around 7.5 s, but there were between-match variations of more than 3 s and high CoVs around 10%. Entropy values show a higher entropy for the distribution of vertical lines, whereas diagonal lines tend to display a more regular distribution, both with only small variations between matches.

### Football RQA

**Figure 4:**
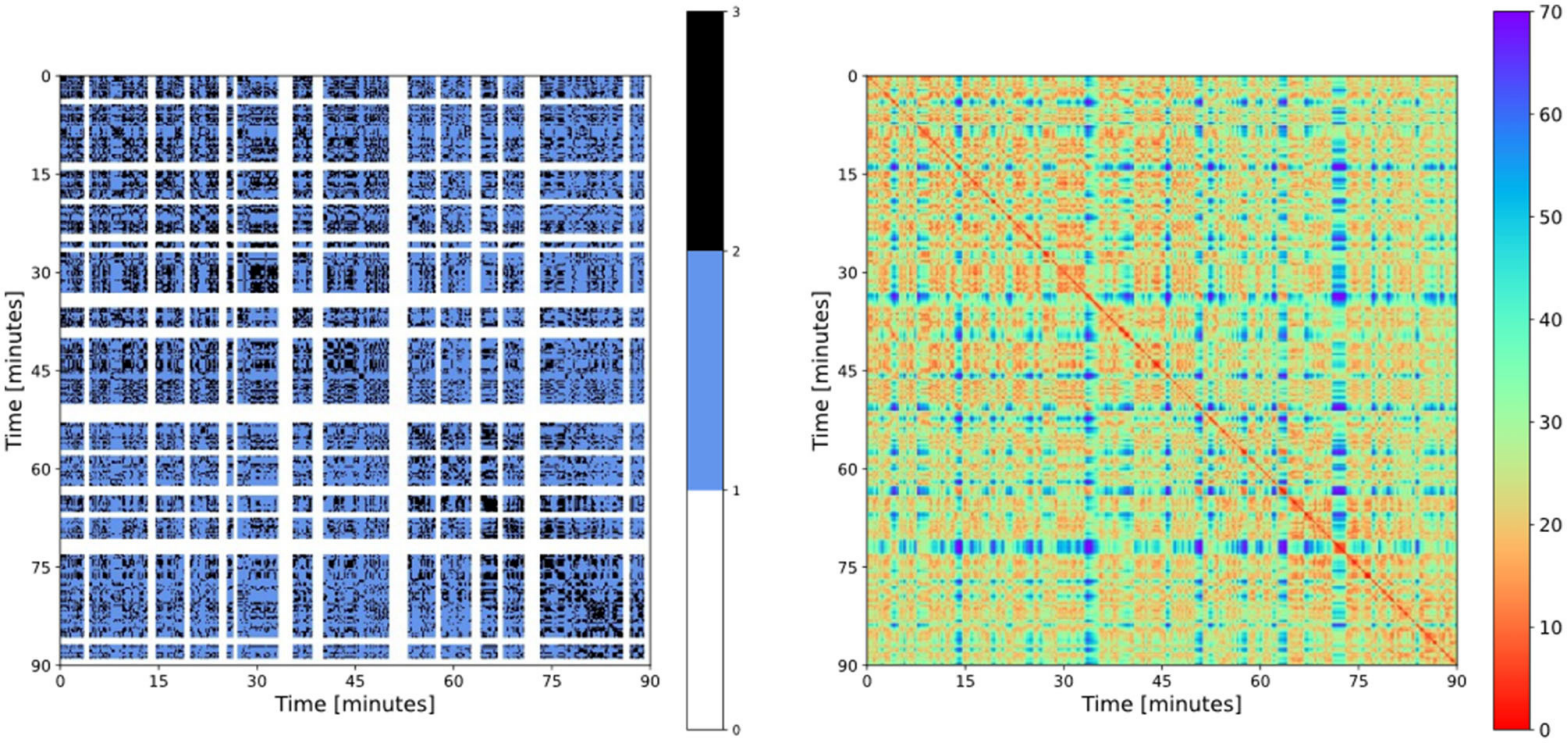
**(Left)** RP with recurrent phases of open play provided by the pattern detection algorithm; **(Right)** color-coded RP of the same match.

Figure 4 (left) shows the result of our pattern detection routine as it identified phases of open play. As mentioned in the methods section, black dots marked here indicate recurrence points with a recurrence threshold of 18 m. This threshold was applied specifically for detecting phases of open play. The original color-coded RP of this match is depicted on the right of Figure 4 to demonstrate the internal functioning of our pattern detection algorithm as well as to allow for comparisons.

Table 2 provides the descriptive statistics of football-specific recurrence parameters. The rate of open play, i.e., the detected patterns, make up 62.2% of the RPs on average with high match-specific variation (range: 34.1–82.9%; CoV: 18.92%). This means that FRP-1 addresses specific properties of a football match. Open play is made up of more stable situations as well as more “chaotic” ones. This specific combination is expressed in FRP-2, with a mean of 31.9% and a range from 28.0 to 40.3%. It is interesting to note that less than half of open play periods are highly recurrent, and again, there is considerable variation between matches. The recurrence share of open play at all recurrence points during a match is, on average, quite high (82.8%), but it also varies considerably between matches (range: 57.7–95.6%).

**Table 2:** Descriptive statistics of the 21 football matches, including the coefficient of variation (CoV) for football-specific recurrence parameters.

**Parameter**	**Description**	**Mean**	**STD**	**Min**	**Max**	**CoV**
FRP-1	Prevalence of open play	0.622	0.118	0.341	0.829	18.92
FRP-2	Recurrence rate in open play	0.319	0.032	0.280	0.403	10.05
FRP-3	Recurrence share of open play	0.828	0.092	0.577	0.956	11.06

### Correlations Between Recurrence Parameters

Table 3 shows the inter-correlations between our 10 recurrence parameters. Recurrence rate (RR) is only weakly correlated with the other recurrence parameters, except for average diagonal line length (LL; strong effect), trapping time (TT) and the three football-specific parameters (medium effects).

**Table 3:** Inter-correlations between traditional and football-specific recurrence parameters for *n* = 21 matches (*significant correlations; **highly significant correlations).

	**RR**	**DET**	**LAM**	**LL**	**TT**	**ENTR**	**ENTR-V**	**FRP-1**	**FRP-2**	**FRP-3**
RR	1	0.015	0.145	−0.565**	−0.423	0.097	−0.089	0.414	0.456*	0.421
DET	0.015	1	0.980**	0.728**	0.809**	0.967**	0.953**	−0.771**	−0.042	−0.749**
LAM	0.145	0.980**	1	0.631**	0.720**	0.956**	0.931**	−0.708**	−0.018	−0.692**
LL	−0.565**	0.728**	0.631**	1	0.953**	0.665**	0.771**	−0.814**	−0.453*	−0.852**
TT	−0.423	0.809**	0.720**	0.953**	1	0.737**	0.825**	−0.821**	−0.357	−0.839**
ENTR	0.097	0.967**	0.956**	0.665**	0.737**	1	0.968**	−0.760**	−0.029	−0.752**
ENTR-V	−0.089	0.953**	0.931**	0.771**	0.825**	0.968**	1	−0.822**	−0.127	−0.813**
FRP-1	0.414	−0.771**	−0.708**	−0.814**	−0.821**	−0.760**	−0.822**	1	0.255	0.961**
FRP-2	0.456*	−0.042	−0.018	−0.453*	−0.357	−0.029	−0.127	0.255	1	0.419
FRP-3	0.421	−0.749**	−0.692**	−0.852**	−0.839**	−0.752**	−0.813**	0.961**	0.419	1

We consistently see very high correlations between recurrence parameters that address dynamic recurrence (diagonal lines) and the corresponding parameters for static recurrence (vertical lines). This finding corresponds to the patch-like patterns of recurrence points found in the football RPs, which feature vertical as well as diagonal lines.

The very high correlations between classical entropy recurrence parameters and determinism and laminarity are unsurprising, as entropy and the frequency of longer recurrent episodes are similar in that they both deal with aspects of line length distribution.

A surprising observation is the negative correlations between the football-specific recurrence parameters and nearly all of the traditional ones. This means that the vast majority of events contributing to the traditional parameters are not from open play but from set plays. In turn, this underlines the importance of defining specific recurrence parameters for open play that would otherwise be missed. The correlation of *r* = 0.961 between the share of open play in the overall match and the share of recurrence points in open play at all recurrence points indicates that there is a relatively constant ratio between recurrence points in both variables. Nevertheless, in both variables, we find that each individual match has an influence on the overall correlation. This relationship warrants further inspection.

In Table 4, the inter-correlations between all of the recurrence parameters and the selected PIs are provided. At first glance, there are several significant and even highly significant correlations with medium to large effect sizes, suggesting the recurrence parameters truly describe relevant aspects of the game.

**Table 4:** Inter-correlations between the recurrence parameters and the traditional PIs for *n* = 21 matches (*significant correlations; **highly significant correlations).

**Performance indicator**	**RR**	**DET**	**LAM**	**LL**	**TT**	**ENTR**	**ENTR-V**	**FRP-1**	**FRP-2**	**FRP-3**
Goals	−0.622**	−0.209	−0.283	0.082	−0.008	−0.210	−0.144	−0.166	−0.293	−0.166
Goal Diff	−0.417	−0.175	−0.171	−0.015	−0.074	−0.218	−0.054	0.035	−0.051	0.110
Shots on goal	−0.334	0.263	0.203	0.472*	0.309	0.331	0.305	−0.444*	−0.244	−0.569**
Distance covered M	0.274	−0.382	−0.383	−0.429	−0.403	−0.295	−0.452*	0.367	−0.048	0.265
Distance covered difference	0.032	0.015	−0.059	0.034	0.111	0.016	0.004	0.203	0.282	0.193
Passes	0.194	−0.375	−0.326	−0.525*	−0.404	−0.454*	−0.446*	0.543*	0.264	0.611**
Passes completed	0.180	−0.358	−0.326	−0.514*	−0.406	−0.418	−0.412	0.572**	0.321	0.644**
Pass% M	0.099	−0.242	−0.259	−0.360	−0.318	−0.252	−0.249	0.519*	0.371	0.581**
Possession difference	0.444*	−0.085	0.036	−0.312	−0.340	−0.045	−0.090	0.382	0.338	0.369
Duels won difference	0.180	−0.391	−0.357	−0.425	−0.393	−0.321	−0.348	0.515*	0.250	0.455*
Corners	0.191	0.312	0.292	0.264	0.218	0.474*	0.386	−0.283	−0.179	−0.385
Stoppages	−0.138	0.245	0.189	0.422	0.276	0.309	0.248	−0.346	−0.250	−0.470*

We found examples where the frequency of events is correlated with the recurrence parameters, for example, the number of goals has a highly negative correlation with the recurrence rate (*r* = −0.622**) and the number of corners is significantly related to entropy; this indicates that there is more ordered behavior surrounding this event. All passing PIs (the number of passes, completed passes, and pass percentage) show significant correlations with the football-specific parameters FRP-1 and FRP-3, both suggesting the prevalence of open play, which is to be expected. This does not hold true for FRP-2, the recurrence rate of open play, as it seems to be an independent variable. There is also the significant negative correlation between the mean distance covered and the entropy of the vertical lines, the latter representing a more static match.

Compared to the frequency of events, the differences in performance indicators between the two teams have a lower impact on the recurrence parameters, for example, the goal difference and the difference in distance covered by the two teams. The difference in possession is significantly correlated with the recurrence rate, suggesting that when a game is dominated by one team, it shows more recurrent patterns. The difference in the percentage of duels won, also representative of one team being dominant, is significantly associated with the two football-specific recurrence parameters (prevalence of open play and the share of recurrence points of open play). This finding is plausible, as the more dominant team has the ability to continue the game by winning more duels than their opponent.

There are contradictory findings in the correlations of the number of shots on goal. They are associated with a lower share of open play, yet we observed a higher average line length, which is representative of more open play. In sum, Table 4 provides interesting insights into the relationships between the recurrence parameters and the traditional PIs. However, these relationships are not yet fully understood.

## Discussion

### Discussion of Methods

One of the aims of this paper was to demonstrate the applicability of recurrence analysis to football matches. In performance analysis, it is a common and effective research strategy to adopt theories and tools from other disciplines to solve specific problems. For example, relative phase, a tool originally used for analyzing inter-limb coordination in movement science (Kelso, 1995), was adapted to investigate the coupling of two tennis players (Palut and Zanone, 2005) and has since found many other applications in performance analysis. The fundamental reason this research strategy is effective is due to the nature of team and net sports. When we consider them as dynamical interaction systems with emergent behaviors, their analysis requires more elaborate tools than the typical approaches that mostly operate with linear models and were borrowed from “normal” behavioral sciences. Therefore, borrowing or importing tools from the field of complex or dynamic systems theory, as we do here with recurrence analysis, is an appropriate and successful strategy in performance analysis.

Ever since the routine introduction of positional data in football about a decade ago, the ideal conditions for recurrence analysis have been established. There are several methodological decisions to be made prior to conducting an analysis. The phase space is quite naturally given in 40 dimensions (2 teams, 10 field players, x and y coordinate), comprehensively representing the spatio-temporal properties of collective behavior. Goalkeepers are excluded because of their atypically steady movements, which would lead to an artificial increase of recurrence.

The matches are truncated to a uniform length of 2 × 45 min without considering extra time. This was done to facilitate comparisons between matches and recurrence parameters. However, it is possible to analyse recurrence in a non-truncated match if there is only a single match of interest. Special care must be taken with game events such as substitutions and red cards.

The decision not to embed the trajectories in a higher dimensional phase space, as is typical in many other applications of recurrence analysis, is justified when we consider the phase space as a comprehensive description of the movement behavior in football. This is also advantageous, as the distances between points on the trajectory obtained with the Euclidean norm are a concrete entity—the average distance of all of the field players' x,y-positions in two points in time.

RPs with color-coded distances are an appropriate visualization of recurrence patterns. Obviously, the color choice was arbitrary in the present study, and future efforts could result in even more impressive, visually-appealing RPs. The application of traditional recurrence parameters in football RPs is not only meaningful in the performance analysis of football, but also in other sports. These parameters allow for quantitative comparisons with other processes, for example, RPs from other sports or from reference processes like white noise, chaotic, or deterministic processes.

The abovementioned research strategy to import tools or theories from other scientific areas may offer an opportunity to improve or extend the imported tool by including specific features of interest. This led us to specify three football-specific recurrence parameters with the aim of further analyzing structures that may become obvious in the RPs of football matches, namely the block-like structures that are associated with longer phases of open play. The definitions of these parameters require the selection of some thresholds for the pattern detection algorithm. Although these selections are conventions and are not grounded in theoretical assumptions, the results suggest that we arrived at a valid model, allowing us to detect the targeted behavior.

### Discussion of Results

As one of the few studies applying recurrence analysis to football, our results are also the first to present color-coded RPs of football matches. The RPs offer a good description of the dynamics of football matches, despite the relative simplicity of recurrence. These dynamic features include the frequency, duration, and distribution of repetitive elements of the game that are associated with set plays and open play. RPs give a comprehensive overview of recurrence in football matches, as they allow for the qualitative characterization of repetitive structures throughout a match. Comparisons between the RPs from different matches support the notion of these distinctive between-match characteristics.

The traditional recurrence parameters were able to describe specific aspects of the football matches and translated well in terms of practical categories of match analysis, e.g., ENTR and ENTR-V: Was a specific match more random than another, with mostly point-like recurrent features, or were there longer and more structured periods? With the findings of this study, we have identified the typical characteristics of the different recurrence parameters for football matches. As a result, the next step is to establish statistical norms and to introduce these recurrence parameters as a new family of performance indicators.

The newly-defined football-specific recurrence parameters successfully characterize unique aspects of football matches. Their correlations with the other recurrence parameters suggest that the new variables do indeed represent these aspects of the matches, with some commonalities with the traditional recurrence parameters. This finding supports the introduction of these three football-specific recurrence parameters as recurrence-based performance indicators in football.

The correlations between the traditional performance indicators and the 10 recurrence parameters introduced in this paper provide insight into their internal relationships. There are many associations between traditional and recurrence-based PIs, and despite the preliminary nature of these initial findings, these associations justify introducing recurrence-based PIs in their own right. In more detail, we found associations between the frequency of events and the recurrence parameters. For example, a high recurrence rate is associated with fewer goals, which implies that goals are unique events and a player must do something special, something that has not happened previously, to score. As another example, the number of longer diagonal lines may increase during set plays because they are being executed in a similar manner, which is shown by the positive relationship between corners and entropy. Again, this supports the notion that football-specific recurrence parameters can be identified from their specific patterns of correlation with traditional PIs. The rather low correlations between recurrence-based variables and the traditional PIs, which are shown by the differences between the teams' performances, could give rise to different approaches to recurrence analysis, such as focusing more on team interaction. These new approaches and their potential to contribute to performance analysis will be presented in the outlook section below.

When introducing new testing instruments, we are reminded by Campbell and Fiske (1959) to ensure that both concurrent and discriminant validity are considered. Based on our findings, these new recurrence parameters have demonstrated the potential to enhance the existing diagnostic repertoire, improving upon both the existing parameters, as well as the traditional performance indicators in football.

As we used the same recurrence threshold (RT = 9 m) and minimum line length (l_min_ = 3) as Stöckl et al. (2017), a comparison was possible despite the fact that the matches occurred 10 years apart. The comparison reveals that, on one hand, the recurrence parameters show a specific range of values for football matches, but on the other hand, modern football matches appear less recurrent and are characterized by shorter recurrent periods: the recurrence rate drops from 2.3 to 1.5%. The share of lines longer than l_min_ decreases from 96.3 to 86.7% for DET (diagonal lines) and from 98.4 to 93.3% for LAM (vertical lines). Contrary to these findings, there is an increase in the mean duration of longer recurrent periods, i.e., the average line length of lines longer than l_min_ for diagonal lines (LL, “prediction time”), increases from 5.5 to 7.5 s, and for vertical lines (TT, “trapping time”), from 6.4 to 8.5 s. When considered together, these findings would imply that modern football has become less recurrent and more “hectic,” but once a stable state is reached, teams manage to remain in controlled states for longer than before. This interpretation could be confirmed in a future study by analyzing the football-specific recurrence parameters during open play. Moreover, as in both cases in our comparison, only the matches between a specific team and their opponents were analyzed, this could have introduced some bias regarding their preferred playing styles.

## Conclusions

As implied by the title of the paper, this study is meant to serve as an initial exploration of the feasibility of recurrence analysis in football. The aims were to increase the number of investigated football matches, to define new football-appropriate recurrence parameters and to validate recurrence parameters against traditional PIs. Having gained insights into recurrence analysis from the present study, there is now the possibility for many future research directions.

It would be beneficial to conduct additional studies using the same methods as presented, but with larger and more diverse match samples, where the matches of many teams are included. Moreover, additional recurrence-based PIs beyond the three defined in this study could be identified to further investigate other unique aspects of football matches. It would also be of interest to study female and youth football contexts. The application of recurrence analysis could produce insightful RPs for other team sports like ice and field hockey, basketball, handball, rugby, and American football. Besides extending the match sample size, different types of samples could also be analyzed. In the present study, only match-based recurrence parameters were presented. In the future, an RQA based on a team's position data could provide team-based parameters or even parameters for individual players based their trajectory.

There are also other approaches of recurrence analysis, which promise new and relevant insights on football matches. The so-called “cross-recurrence plots” do not have the same entity on the x and y axes but different ones, such as a team's position on the x-axis and the opponent's position on the y-axis. This could contribute to insights into positional team interactions. Unfortunately, the recurrence-based PIs presented so far are equally limited to static summaries of match behavior as traditional PIs, rather than reporting the dynamical change of behavior throughout a match. A potential solution could be to use so-called “windowed recurrence analysis,” where the recurrence parameters are calculated for a specific moving time window. This design would provide recurrence parameters continuous in time reflecting the dynamics of recurrence over an entire match.

Recurrence analysis may be of practical value as well, for example, to support qualitative match analyses using a RP-based analysis tool. This simple application could display the match video from 10 s (or any other convenient time interval) before and after a selected point on the x-axis in the RP, as well the corresponding part of the match on the y-axis. In practice, this would allow a sports analyst to quickly search for interesting points in the RP by having the relevant video clips easily accessible. This also facilitates the retrieval of similar clips which may not be easily found if they were not marked by specific events, such as during set plays. Another practically supportive option would be to filter by specific match characteristics, such as only analyzing recurrence behavior when one team has ball possession. The analysis and comparison of recurrence structures during small-sided games could inform the design of training exercises.

From the perspective of data analytics, automated RP comparisons based on deep neural networks would be an interesting advancement of recurrence analysis. If applied in an unsupervised mode, this technology could cluster similar matches, but in a supervised mode, the algorithm could be trained using a specified training set to automatically classify future matches.

The current paper has demonstrated the feasibility of using recurrence analysis for football matches, as well as introduced both general and football-specific recurrence parameters with consideration for discriminant and concurrent validity compared to traditional PIs. The addition of recurrence analysis as a tool for analyzing football matches introduces the opportunity for new methods for both theoretical and practical performance analysis. Maybe in the near future, when recurrence parameters will be widely acknowledged in the large family of PIs in performance analysis, a match report in the Monday's issue of a newspaper will read like this: “Despite good determination at 90% and fantastic entropy of over 3, the home team did not manage to create many goal scoring opportunities because their gameplay was too static, as shown by the high laminarity and high recurrence rates overall and specifically during open play!”

## Data Availability Statement

The original contributions presented in the study are included in the article/supplementary material, further inquiries can be directed to the corresponding author/s.

## Author Contributions

ML and HM contributed to conception and design of the study. RP organized the database. SH performed the data processing and calculation of recurrence statistics. HM developed the pattern detection algorithms. ML wrote the first draft of the manuscript. RP, SH, and HM wrote sections of the manuscript. All authors contributed to manuscript revision, read, and approved the submitted version.

## Conflict of Interest

RP was employed by the company Kinexon GmbH. The remaining authors declare that the research was conducted in the absence of any commercial or financial relationships that could be construed as a potential conflict of interest.

## Publisher's Note

All claims expressed in this article are solely those of the authors and do not necessarily represent those of their affiliated organizations, or those of the publisher, the editors and the reviewers. Any product that may be evaluated in this article, or claim that may be made by its manufacturer, is not guaranteed or endorsed by the publisher.
